# P-218. Examining CD4, Viral Load and STI Co-Infection as Predictors of Severe Mpox Outcomes Among a Cohort of People with HIV in New York City

**DOI:** 10.1093/ofid/ofaf695.440

**Published:** 2026-01-11

**Authors:** Ofole Mgbako, Alex J Pelliccione, Jacob McLean, Asa Radix, Mark N Sayegh, Rustin A Zomorodi, Jonathan Berardi, Justin Chan, Madeline DiLorenzo, Robert Pitts

**Affiliations:** NYC Health+Hospitals, Brooklyn, NY; NYU Langone Health, Brooklyn, NY; New York Presbyterian - Columbia University Irving Medical Center, New York, New York; Callen-Lorde Community Health Center, New York, New York; NYC Health + Hospitals/ Harlem Columbia University, scarsdale, New York; Icahn School of Medicine at Mount Sinai, New York, New York; Rutgers New Jersey Medical School, Newark, New Jersey; NYU Grossman School of Medicine, New York City, New York; NYU Langone Health, Brooklyn, NY; NYU Langone Health, Brooklyn, NY

## Abstract

**Background:**

HIV infection with low CD4 count is a known risk factor for severe clinical outcomes in patients with mpox in the current global mpox outbreak. However, it is unclear how a low CD4 in addition to other clinical factors, such as viremia or co-infection with other sexually transmitted infections (STIs), serve as predictors of mpox severity. We sought to assess the risk of mpox-related hospitalization in patients with HIV, examining CD4, viral load (VL), antiretroviral treatment (ART) prescription as a proxy of adherence, and STI co-infection in people diagnosed with mpox in New York City (NYC).

**Table 1.**
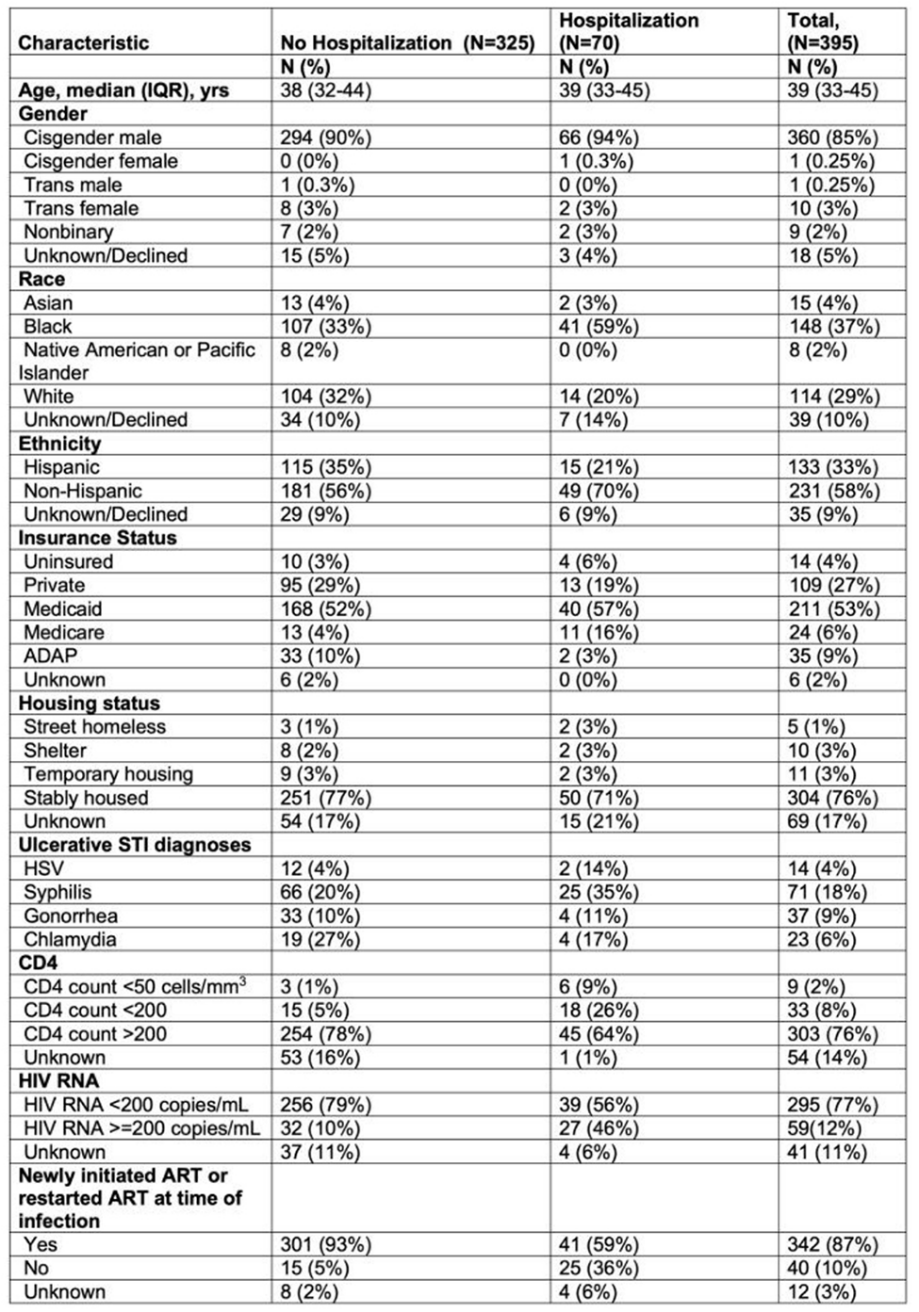
Demographics and Clinical Characteristics of Patients with HIV and Mpox Receiving Tecovirimat in NYC by Hospitalization Status (N=395)

**Table 2.**
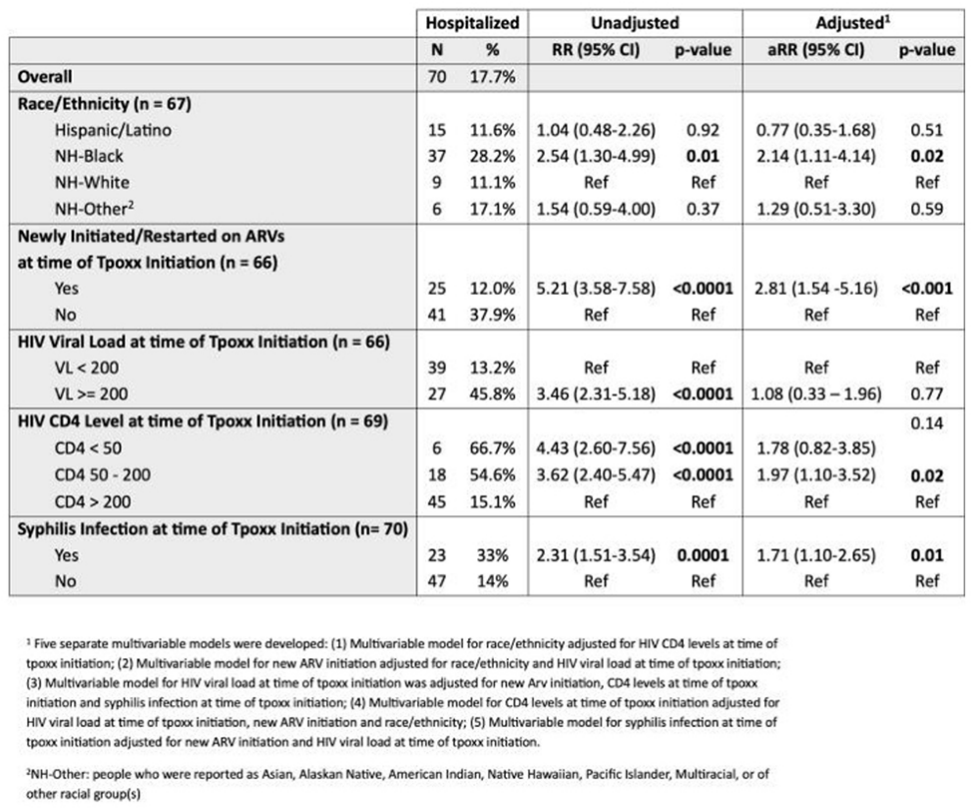
Relative Risk of Hospitalization in People with HIV and Mpox Receiving Tecovirimat in NYC

**Methods:**

We analyzed retrospective deidentified data from 8 NYC healthcare systems for patients with mpox and HIV initiating tecovirimat, an antiviral used for severe mpox, from May–December 2022. We used descriptive statistics to assess demographic and clinical characteristics of patients who were hospitalized. We used multivariable Poisson regression with robust standard errors to examine factors associated with hospitalization, adjusting for potential confounders.

**Results:**

Of 395 patients with HIV and mpox who initiated tecovirimat, 17.72% (N=70) were hospitalized. Demographics, CD4, VL and ART prescription data are summarized in Table 1. Hospitalized patients were more likely to be non-Hispanic Black, have concurrent herpes or syphilis diagnoses, HIV viremia (HIV RNA >=200copies/mL) or CD4 < 200 cells/mm^3^. Multivariable models (Table 2) show patients who were non-Hispanic Black (aRR=2.14), who newly initiated or restarted ART at the time of mpox diagnosis (aRR=2.81), who had CD4 50-200 cells/mm^3^ (aRR=1.97), or had syphilis (aRR=1.71) were at higher risk of hospitalization after adjusting for confounding variables.

**Conclusion:**

In this diverse NYC cohort across multiple health systems, our findings highlight the importance of addressing structural racism as well as co-management of HIV and other STIs alongside acute mpox infection. Limitations of our analysis include lack of data on mpox vaccination status, few patients with CD4 < 50, and use of ART prescription as an imperfect proxy for ART adherence.

**Disclosures:**

Ofole Mgbako, MD, MS, Gilead Sciences: Advisor/Consultant Robert Pitts, MD MPH, Gilead Inc: Advisor/Consultant|ViiV: Advisor/Consultant

